# Retraction: Protective role of taurine against arsenic-induced mitochondria-dependent hepatic apoptosis via the inhibition of PKCδ-JNK pathway

**DOI:** 10.1371/journal.pone.0324225

**Published:** 2025-05-12

**Authors:** The PLOS One Editors

After this article [1] was published, concerns were raised about Figures 2, 4–6, 8, and 10. Specifically, concerns were identified within the figures of [1] as follows:

- There appear to be vertical discontinuities in Figure 4B;
- There appear to be similarities between parts of the two Liver – ‘Phospho ERK1/2’ panels in Figure 6C;
- There appear to be multiple regions of similarity within the As+Tau panel, and between the As+Tau and the Normal panel of Figure 10;

Additionally, when aspect ratios and/or orientation are adjusted, there appear to be similarities between panels or parts of panels in [1] and in other articles published by the same authors, including:

- The ‘As treated’ panel of Figure 2C, the ‘Hg’ panel in Figure 4A of [2], the ‘Cd (a)’ panel in Figure 2A of [3], and the ‘APAP treated’ panel in Figure 3E of [4].
- The lower liver - ‘Phospho ERK1/2’ panel in Figure 6C and the Liver – ‘ERK1/2’ panel of Fig 3A in [5].

The first author JD stated that work for different projects was conducted simultaneously in the laboratory and the similarities noted between Figures 2 and 6 and other articles published by the same group were the result of errors made during figure preparation. They stated that the original underlying images and raw data are not available.

In the absence of the original images, the above concerns remain unresolved and the *PLOS One* Editors retract this article.

It was additionally noted that there appear to be similarities between the ‘Phospho PKCδ’ panel in Figure 8B and the ‘Keap1’ panel of Figure 12 in [6], which was published later by different authors.

The ‘As treated’ panel in Figure 2C reports material from [4], published in February 2010 by Taylor & Francis, and the lower Liver – ‘Phospho ERK1/2’ panel in Figure 6C reports material from [5], published in May 2010 by Elsevier, which are not offered under a CC BY license and are therefore excluded from this article’s [1] license.

JD, PM, and PCS did not agree with the retraction. JG either did not respond directly or could not be reached.
